# Nuclear factor kappa B in urine sediment: a useful indicator to detect acute kidney injury in *Plasmodium falciparum* malaria

**DOI:** 10.1186/1475-2875-13-84

**Published:** 2014-03-07

**Authors:** Chuchard Punsawad, Parnpen Viriyavejakul

**Affiliations:** 1 School of Medicine, Walailak University, 222 Thasala District, Nakhon Si Thammarat 80161, Thailand; 2 Department of Tropical Pathology, Faculty of Tropical Medicine, Mahidol University, 420/6 Rajvithi Road, Bangkok, Thailand; 3 Center for Emerging and Neglected Infectious Diseases, Mahidol University, Bangkok, Thailand

**Keywords:** Malaria, *Plasmodium falciparum*, Nuclear factor kappa B (NF-κB), Urinary sediment, Acute kidney injury (AKI)

## Abstract

**Background:**

Acute kidney injury (AKI) is one of the major complications of *Plasmodium falciparum* malaria, especially among non-immune adults. It has recently been revealed that activation of transcription factor nuclear factor kappa B (NF-κB) induces pro-inflammatory gene expression involved in the development of progressive renal inflammatory diseases. The aim of this study was to determine whether urinary sediment NF-κB p65 can act as a biomarker for AKI in patients with *P. falciparum* malaria.

**Methods:**

Urinary sediments from malaria patients, including *Plasmodium vivax* malaria, uncomplicated *P. falciparum* malaria, complicated *P. falciparum* malaria without AKI (serum creatinine-Cr <3 mg/dl) and complicated *P. falciparum* malaria with AKI (Cr ≥3 mg/dl) were used to determine NF-κB p65 level by sandwich enzyme-linked immunosorbent assay (ELISA). Urinary sediments obtained from healthy controls were used as a normal baseline. Correlations between levels of urinary sediment NF-κB p65 and pertinent clinical data were analysed.

**Results:**

Urinary sediment NF-κB p65 levels were significantly increased on the day of admission (day 0) and on day 7 post-treatment in complicated *P. falciparum* malaria patients with AKI, compared with those without AKI (*p* = 0.001, *p* <0.001, respectively), *P. vivax* patients (all *p* <0.001) and healthy controls (all *p* <0.001). NF-κB p65 levels in urinary sediment cells showed a significant positive correlation with serum Cr (Day 0: *r*_*s*_ = 0.792; *p* <0.001, Day 7: *r*_*s*_ = 0.605; *p* <0.001) and blood urea nitrogen (BUN) (Day 0: *r*_*s*_ = 0.839; *p* <0.001, Day 7: *r*_*s*_ = 0.596; *p* <0.001).

**Conclusions:**

Urinary sediment NF-κB p65 level is a useful indicator for estimating renal tubular epithelial cell damage and subsequent development of AKI among patients with *P. falciparum* malaria.

## Background

Acute kidney injury (AKI) is one of the serious complications of *Plasmodium falciparum* infection, with mortality rates as high as 70% among untreated patients [1]. The rare incidence has also been reported in *Plasmodium vivax*, occasionally contributing to renal impairment [2]. Malarial AKI is more common among adults in tropical areas where transmission of malaria is low or unstable and where the disease occurs at all ages [3]. Clinical presentations of malarial AKI patients are usually oliguria with increasing levels of serum creatinine (Cr), but urine output may also be normal [3]. Several factors have been implicated in the pathogenesis of malarial AKI, including the cyto-adherence of parasitized red blood cells (PRBCs), the effect of various chemical mediators, dehydration, intravascular haemolysis, and hyperparasitaemia [4]. Studies on Southeast Asian adults dying from severe falciparum malaria have indicated that the frequency of PRBC sequestration in the renal blood vessels of patients dying from AKI was significantly higher than those without AKI [5,6].

AKI is generally characterized by the sudden loss of the kidneys’ ability to concentrate urine, excrete waste, conserve electrolytes, and maintain fluid balance. The disease is associated with a mortality rate of between 50 and 80% [7]. The loss of kidney function is usually detected by the measurement of serum Cr or by a rise in blood urea nitrogen (BUN). BUN is a non-specific indicator of renal function as urea production can be altered by dehydration, food intake and tissue catabolism [3]. Thus, serum Cr is more specific in assessing renal function than BUN [8]. In general, it has been approximated that the incidence of AKI in the intensive care unit was about 1-25% and the mortality rate was 15-60% [9]. In Thailand, the incidence of AKI in *P. falciparum* malaria varies from 0.14-0.18% at the Hospital for Tropical Diseases, Bangkok [10] and up to 73.9% at Mae Sot General Hospital using RIFLE criteria (risk, injury, failure, loss, and end-stage kidney disease) [11]. Existing kidney markers such as serum Cr, urine output, and estimated glomerular filtration rate, become elevated only when a significant damage or loss of kidney function has occurred. Recently, urinary biomarkers such as interleukin-18 (IL-18), kidney injury molecule-1 (KIM-1) and neutrophil gelatinase-associated lipocalin (NGAL) have been reported as non-invasive and relatively inexpensive tools for assessing the degree and characteristics of inflammation and tubulo-interstitial damage [12,13]. Several studies have demonstrated that nuclear factor-κB (NF-κB) is activated in the renal tubular epithelial cells, glomeruli and urothelial cells in renal injury [14,15] and that renal inflammation can occur after induction by a variety of stimuli [14,15]. NF-κB is a family of dimeric transcription factors that regulates the expression of numerous genes involved in inflammation and cell proliferation [16]. The increased activation of NF-κB may indicate a response to renal tubular injury [14,15].

This study investigated NF-κB p65 levels in urinary sediments from malaria patients to assess whether urinary sediments NF-κB p65 can serve as a sensitive tool for detecting AKI in *P. falciparum* malaria patients.

## Methods

### Subjects

Thirty-nine malaria patients admitted to the Hospital for Tropical Diseases, Faculty of Tropical Medicine, Mahidol University, Thailand were enrolled into this study. Classification of malaria species was based on microscopic identification. The patients were divided into four groups: 1) *P. vivax* malaria (n = 11), 2) uncomplicated *P. falciparum* malaria (n = 8), 3) complicated *P. falciparum* malaria without AKI (Cr <3 mg/dl) (n = 10), and, 4) complicated *P. falciparum* malaria with AKI (Cr ≥3 mg/dl) (n = 10). Classification of complicated *P. falciparum* malaria was based on WHO criteria [3]. The control group consisted of 14 healthy volunteers living in Bangkok, a non-endemic malaria area. This group had no history of malaria infection. This study was approved by the Ethics Committee, Faculty of Tropical Medicine, Mahidol University (MUTM 2010-035-01, MUTM 2010-035-02). Written informed consent was obtained from all patients or their legal representatives before enrollment into the study.

### Blood and urine samples

Five ml of whole blood were obtained from malaria patients on day 0 (pre-treatment) and day 7 (post-treatment). Samples of clotted blood were centrifuged at 1,700 g for 10 min and the serum was harvested and stored in an aliquoted state at -70°C. The serum was used to measure Cr and BUN levels to investigate kidney function. Urinary sediment NF-κB p65 was determined by enzyme-linked immunosorbent assay (ELISA). Early morning whole-stream urine specimens were collected from all subjects on day 0 (pre-treatment) and day 7 (post-treatment). For urine sediment preparation, urine samples were centrifuged at 3,000 g for 15 min and urinary cell pellets were collected and stored at -70°C until use. The proteins from urinary cell pellets were extracted by lysis buffer (Cell Signaling, MA, USA) containing protease inhibitors (Sigma-Aldrich, MO, USA) according to the manufacturers’ protocol. In summary, the pellets were sonicated on ice, centrifuged at 14,000 g for 10 min at 4°C and the supernatants were harvested and used for ELISA. Protein concentrations were determined by Bradford assay (Pierce Biotechnology, IL, USA), using bovine serum albumin (BSA) as the standard. Fifteen ml of urine sample were sent to the laboratory immediately or stored at 4°C overnight for urine analysis.

### Measurement of urinary sediment NF-κB p65 levels

The NF-κB p65 levels in urinary sediments were measured by sandwich ELISA kit (Cell Signaling, MA, USA) according to the manufacturer’s protocol. Phospho-NF-κB p65 mouse monoclonal antibody diluted to 1: 100 in PBS was coated on a 96-well microplate and stored overnight. Protein samples (45 μg/well) were added and incubated for two hours at 37°C. The plate was then washed with PBS containing 0.05% (w/v) Tween-20 (PBS-T). Phospho-NF-κB p65 rabbit monoclonal antibody was added to the wells and incubated for one hour at 37°C to detect the captured phospho-NF-κB p65 protein. After washing with PBS-T, anti-rabbit IgG secondary antibody conjugated with horseradish peroxidase (HRP) was added and incubated for 30 min at 37°C. The colour reaction was developed by incubation with 3,3′,5,5′ tetramethylbenzidine (TMB) substrate solution (Cell Signaling, MA, USA). Finally, the reaction was stopped with 0.18 M sulphuric acid. Optical density (OD) was measured at 450 nm. All assays were carried out in duplicate.

### Statistical analysis

Statistical analysis was performed using SPSS version 17.0 software (SPSS, IL, USA). All results were presented as mean ± standard error of the mean (SEM). The normality of distribution was determined by the Kolmogorov-Smirnov test. Differences in levels of urinary sediment NF-κB p65 between groups were compared by Mann-Whitney U-test. Differences in levels of urinary sediment NF-κB p65 within groups between day 0 and day 7 were tested by Wilcoxon signed-rank test. In addition, the correlation between levels of NF-κB p65 and pertinent clinical data including age, malaria parasite density, haemoglobin, haematocrit, white blood cells (WBC) and urinary laboratory parameters were calculated using Spearman’s rank correlation (*r*_*s*_). A *p* value <0.05 was considered statistically significant.

## Results

### Clinical and laboratory data of malaria patients

Clinical data of the malaria patients and healthy controls are shown in Table 1 and the results of urinary analysis are presented in Table 2. On admission, the mean malaria parasite density in the group of complicated *P. falciparum* malaria patients without AKI (Cr <3 mg/dl) (670,124 parasite/μl) was similar to those with AKI (Cr ≥3mg/dl) (547,766 parasite/μl) (*p* = 0.602). At day 7 post-treatment, no asexual form of malaria parasite was found in the peripheral blood of any malaria patient. A significant increase in the level of serum Cr was found in the group of complicated *P. falciparum* malaria patients with AKI (6.3 ± 0.5 mg/dl), compared with complicated *P. falciparum* malaria patients without AKI (1.8 ± 0.2 mg/dl) (*p* = 0.009). The Cr level was 3.5 times higher in malaria patients with AKI. No significant difference in serum Cr was found in *P. vivax* patients (0.8 ± 0.1 mg/dl) and uncomplicated *P. falciparum* patients (0.9 ± 0.1 mg/dl), compared with the healthy controls upon admission and day 7 post-treatment (all *p* >0.05). Serum BUN was also significantly higher in *P. falciparum* malaria with AKI than in *P. vivax*, uncomplicated *P. falciparum* and *P. falciparum* malaria patients without AKI (all *p* <0.05).

**Table 1:** Demographic data and clinical data of malaria patients and healthy controls

	***P. vivax***	***P. falciparum *** **uncomplicated**	***P. falciparum *** **complicated (Cr <3.0 mg/dl)**	***P. falciparum *** **complicated (Cr ≥3.0 mg/dl)**	**Healthy controls**
n	11	8	10	10	14
Age (year)	24 ± 2.6	27 ± 2.8	27 ± 1.3	30 ± 5.9	31 ± 2.4
Sex (male:female)	11:0	8:0	5:0	5:0	7:7
Parasite (μl) D0	20,376^a, b^	249,101^a, b^	670,124^a^	547,766^a^	0
Parasite (μl) D7	0^c^	0^c^	0^c^	0^c^	0
Serum Cr D0	0.8 ± 0.1^b^	0.9 ± 0.1^b^	1.8 ± 0.2^a, b^	6.3 ± 0.5^a^	0.8 ± 0.0
Serum Cr D7	0.8 ± 0.3^b^	0.8 ± 0.6^b^	0.7 ± 0.5^b, c^	8.6 ± 0.2^a, c^	0.9 ± 0.1
Serum BUN D0	13.4 ± 1.0^b^	15.9 ± 2.2^b^	32.6 ± 4.7^b^	109.4 ± 14.0	NA
Serum BUN D7	12.6 ± 0.8^b^	12.4 ± 1.3^b^	12.8 ± 0.8^b, c^	75.6 ± 0.5^c^	NA

^a^Significance of *p* <0.05 compared with healthy controls.

^b^Significance of *p* <0.05 compared with *P. falciparum* malaria associated with AKI (Cr ≥3 mg/dl).

^c^Significance of *p* <0.05 compared with the day of admission.

**Table 2:** Urine analysis of malaria patients and healthy controls

	***P. vivax***	***P. falciparum *** **uncomplicated**	***P. falciparum *** **complicated (Cr <3.0 mg/dl)**	***P. falciparum *** **complicated (Cr ≥ 3.0 mg/dl)**	**Healthy controls**
n	11	8	10	10	14
Specific gravity D0	1.017 ± 0.0^a, b^	1.017 ± 0.0^a^	1.021 ± 0.0^a^	1.021 ± 0.0^a^	1.010 ± 0.0
Specific gravity D7	1.021 ± 0.0^a^	1.015 ± 0.0^b^	1.022 ± 0.0^a^	1.021 ± 0.0^a^	1.010 ± 0.0
pH D0	6.8 ± 0.3^b^	6.1 ± 0.2^b^	5.6 ± 0.3^a, b^	5.0 ± 0.0^a^	6.6 ± 0.2
pH D7	5.6 ± 0.2^a, b, c^	6.0 ± 0.3	6.2 ± 0.1	6.2 ± 0.1^c^	6.3 ± 0.2
Urine protein D0	27.3 ± 9.8^a, b^	43.8 ± 12.3^a, b^	62.0 ± 17^a^	160.0 ± 56.7^a^	0 ± 0.0
Urine protein D7	4.5 ± 3.0	3.1 ± 3.1^c^	0 ± 0.0^c^	6.0 ± 4.0^c^	0 ± 0.0
RBC in Urine D0	0.7 ± 0.3^a, b^	0.5 ± 0.3^a, b^	3.4 ± 1.1^a^	11.8 ± 6.4^a^	0 ± 0.0
RBC in Urine D7	0.1 ± 0.1^b^	0 ± 0.0^b^	0 ± 0.0^b, c^	6.4 ± 3.9^a^	0 ± 0.0
WBC in Urine D0	0.8 ± 0.3^b^	1.6 ± 0.5^b^	1.6 ± 0.2^a, b^	2.4 ± 0.2^a^	1.0 ± 0.0
WBC in Urine D7	1.1 ± 0.1^b^	1.0 ± 0.0^b^	2.4 ± 0.2^a, b, c^	3.8 ± 0.3^a, c^	1.3 ± 0.2
Epithelium D0	1.1 ± 0.1^b^	1.6 ± 0.3^b^	1.6 ± 0.4^b^	5.0 ± 1.0^a^	1.1 ± 0.1
Epithelium D7	1.1 ± 0.1^b^	1.0 ± 0.0^b^	3.0 ± 0.6^a, b, c^	10.6 ± 1.3^a, c^	1.2 ± 0.2

^a^Significance of *p* <0.05 compared with healthy controls.

^b^Significance of *p* <0.05 compared with *P. falciparum* malaria associated with AKI (Cr ≥3 mg/dl).

^c^Significance of *p* <0.05 compared with the day of admission.

All malaria patient groups showed higher proteinuria than the healthy controls upon admission (all *p* <0.05). Proteinuria was significantly increased in complicated *P. falciparum* (both with and without AKI groups), compared with *P. vivax* and uncomplicated *P. falciparum* (all *p* <0.05) (Table 2). The number of red blood cells (RBC) in the urine was significantly increased in all malaria groups compared with healthy controls upon admission (all *p* <0.05). Significantly lower RBC numbers were observed in *P. vivax* and uncomplicated *P. falciparum,* compared with complicated *P. falciparum* (both with and without AKI groups) (all *p* <0.05). In addition, the mean number of WBC in the urine sediment was highest in the group of complicated *P. falciparum* malaria with AKI compared with the healthy controls (*p* <0.001), *P. vivax*, uncomplicated *P. falciparum* and *P. falciparum* malaria patients without AKI (all *p* <0.05). At day 7, the microscopic levels of RBC, WBC and epithelial cells remained significantly elevated in the urine samples from complicated *P. falciparum* malaria with AKI patients, compared to the healthy controls (*p* = 0.02, *p* = 0.001 and *p* <0.001, respectively) (Table 2).

### NF-κB p65 level in urinary sediment cells

**Figure 1:**
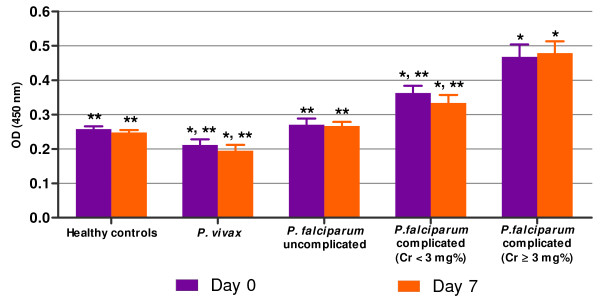
**Levels of NF-κB p65 in the urinary sediment cells of malaria patients and healthy controls.** Protein isolated from the urinary sediment cells was used to measure the level NF-κB p65 by ELISA. ^*^Significance of *p* <0.05 compared with healthy control. ^**^Significant of *p* <0.05 compared with *P. falciparum* malaria associated with AKI (Cr ≥3 mg/dl).

NF-κB p65 levels measured by ELISA in the urinary sediment cells of malaria patients and healthy controls are shown in Figure 1. On admission (day 0), the mean level urinary sediment NF-κB p65 was significantly elevated in complicated *P. falciparum* malaria with AKI (0.47 ± 0.02), compared with *P. vivax* malaria (0.21 ± 0.02), uncomplicated *P. falciparum* malaria (0.27 ± 0.02) and complicated *P. falciparum* malaria without AKI (0.36 ± 0.01), (*p* <0.001*, p* <0.001, *p* = 0.001, respectively). Compared with the healthy controls (0.26 ± 0.01), the level of urinary sediment NF-κB p65 increased significantly in the groups of complicated *P. falciparum* malaria with and without AKI (all *p* <0.001). However, *P. vivax* malaria patients showed a significant decrease in urinary sediment NF-κB p65 level compared with healthy controls (*p* = 0.019), while NF-κB p65 levels in uncomplicated *P. falciparum* malaria patients were similar to the healthy controls (*p* >0.05).

On day 7 post-treatment, the NF-κB p65 levels in the urinary sediment cells were significantly increased in both groups of complicated *P. falciparum* malaria patients (without AKI = 0.33 ± 0.02; with AKI = 0.48 ± 0.02), compared with the healthy controls (0.25 ± 0.01; *p* = 0.001, *p* <0.001, respectively). Among the complicated *P. falciparum* malaria, AKI group showed significantly higher urine NF-κB p65 levels, compared to those without AKI (*p* <0.05). In addition, the level of urinary sediment NF-κB p65 remained significantly decreased in patients infected with *P. vivax* malaria (0.19 ± 0.02), compared with the healthy controls (*p* = 0.014). The lowest levels of urinary sediment NF-κB p65 were found in *P. vivax* malaria patients. However, no significant differences in the levels of urine NF-κB p65 were observed between day 0 and day 7 in all malaria patient groups (all *p* >0.05).

### Correlations between urinary sediment NF-κB p65 level and clinical data

**Figure 2:**
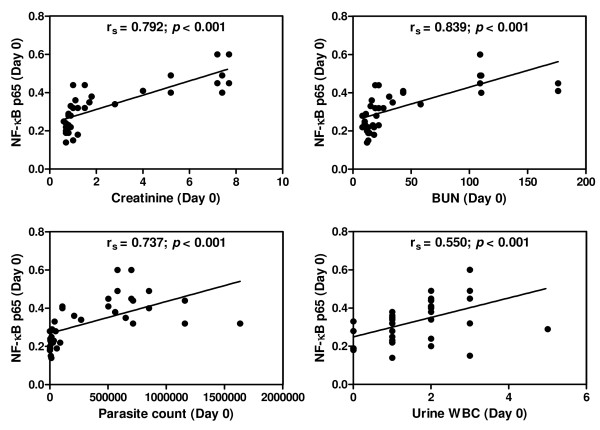
**Correlations between urinary sediment NF-κB p65 and clinical data on the day of admission (n = 39).** Data analysed by Spearman’s rank correlation.

**Figure 3:**
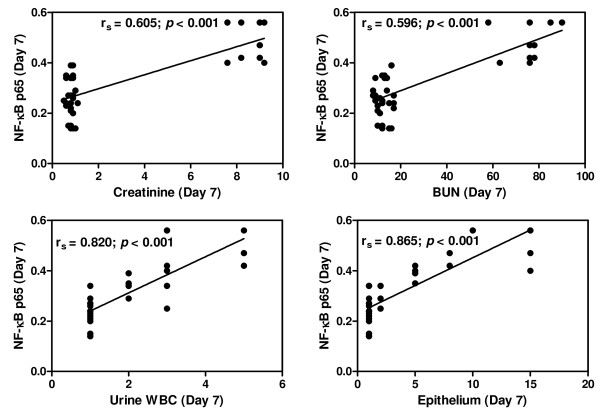
**Correlations between urinary sediment NF-κB p65 and clinical data on day 7 post-treatment (n = 39).** Data analysed by Spearman’s rank correlation coefficient.

On admission, NF-κB p65 levels in the urinary sediment cells showed significant positive correlations with serum Cr (*r*_*s*_ = 0.792; *p* <0.001), BUN (*r*_*s*_ = 0.839; *p* <0.001), parasite density (*r*_*s*_ = 0.737; *p* <0.001) and microscopic WBC (*r*_*s*_ = 0.550; *p* <0.001) (Figure 2). On day 7 post-treatment, NF-κB p65 showed significantly positive correlations with serum Cr (*r*_*s*_ = 0.605; *p* <0.001), BUN (*r*_*s*_ = 0.596; *p* <0.001), microscopic WBC (*r*_*s*_ = 0.820; *p* <0.001) and urinary epithelial cells (*r*_*s*_ = 0.865; *p* <0.001) (Figure 3).

Within the complicated *P. falciparum* malaria with AKI group, a significant positive correlation was found between NF-κB p65 in the urinary sediment cells and serum Cr. on the day of admission (*r*_*s*_ = 0.9000; *p* = 0.037) and on day 7 post-treatment (*r*_*s*_ = 0.975; *p* <0.005). There was no significant correlation between NF-κ B p65 levels in the urinary sediment cells and other urinary parameters including specific gravity, pH and urine protein in any malaria patient group.

## Discussion

The precise mechanism of AKI in *P. falciparum* malaria has not been fully understood. Several hypotheses have been demonstrated including mechanical obstruction by infected PRBCs, immune mediated glomerular pathology, fluid loss due to multiple mechanisms and alterations in renal microcirculation [4,17,18]. The histopathological finding in malaria AKI showed a variable mixture of acute tubular necrosis (ATN), interstitial nephritis and glomerulonephritis. ATN is the most consistent histological finding, presenting with cloudy swelling, hemosiderin granular deposits and cell necrosis [4]. An ultrastructural study of the kidney in fatal falciparum malaria showed PRBC sequestration in the glomerular and tubulo-interstitial blood vessels; and acute tubular damage is an important feature [6]. With regard to chemical mediators, inflammatory cells, particularly monocytes stimulated by glycosylphosphatidylinositol, can enhance the synthesis of various cytokine cascades and mediators. These mediators cause changes in blood volume status, vasodilation and increases vascular permeability leading to hypovolaemia which can contribute to ischemic renal failure [17]. Activation of NF-κB in renal tubular epithelial cells and macrophages in human renal tissues has been reported [14]. In the present study, NF-κB p65 levels in urinary sediment cells were highest and remained significantly elevated from pre-treatment to day 7 post-treatment in complicated *P. falciparum* malaria associated with AKI (Cr ≥3 mg/dl), compared with healthy controls and other malaria groups. It is possible that activation of NF-κB p65 in the urine sediment containing renal epithelial cells and WBC is triggered by various ligands or proteins of malaria parasites that induce up-regulation of the NF-κB p65 signalling pathway. This eventually leads to nuclear translocation of NF-κB and regulation of gene expression involved in inflammation and apoptosis. The inflammation in the interstitium of kidney tissue has been proposed to play a major role in the pathophysiology of AKI [19,20]. A study also reported that proximal tubular epithelial cells are an important source of cytokine production, such as interleukin (IL)-18 and IL-16. These cytokines enter the interstitium and result in activation of inflammatory cells [21].

In this study, ELISA was used to measure NF-κB p65 levels in urinary sediment cells. Preliminary experiments using urine supernatants from malaria patients were unable to detect NF-κB p65. Either NF-κB p65 was not excreted into the urine or the molecule was too diluted within the urine to be measurable directly. Similar finding was documented for urinary haem oxygenese-1 (uHO-1) measurement, where it was undetectable in the urine supernatant [22]. Activation of NF-κB p65 has been demonstrated in glomeruli, in tubulo-interstitial cells, in infiltrating cells of obstructed kidneys [23], as well as in renal tubular cells [14]. The proximal and distal tubular epithelial cells were documented as major NF-κB p65 producers [22]. Recent studies show that NF-κB p65 activation has also been detected in peripheral blood mononuclear cells (PBMCs) from complicated *P. falciparum* malaria patients [24]. The source of NF-κB p65 in urine sediment possibly originated from renal tubular cells, which were detached from the basement membrane into the lumen of renal tubules and/or from the WBC. Both renal epithelial cells and WBC were found upon examination under a light microscope. The NF-κB p65 levels may indicate ongoing renal damage after infection with malaria. Complicated *P. falciparum* malaria without AKI also showed a rise in NF-κB p65 levels, compared to healthy controls, but was significantly lower than AKI group (Figure 1). Data suggest that NF-κB p65 levels may reflect the degree of renal tubular damage caused by PRBC sequestration, leading to restricted local blood flow and host cytokine response, contributing to progressive renal tubular injury.

AKI is usually diagnosed by increased serum Cr or BUN levels. Newer biomarkers have been used for the early diagnosis of AKI, such as IL-18, KIM-1 and NGAL [25]. This study documented a potential role of NF-κB p65 level in urinary sediment cells in assessing AKI in malaria patients. Within the group of complicated *P. falciparum* malaria associated with AKI, a significant positive correlation was found between NF-κB p65 in the urinary sediment cells and serum Cr on the day of admission (*r*_*s*_ = 0.9000; *p* = 0.037) and on day 7 post-treatment (*r*_*s*_ = 0.975; *p* <0.005). However, lower urinary NF-κB p65 levels were detected in *P. vivax* and uncomplicated *P. falciparum* patients. Therefore, NF-κB p65 may be a valuable indicator of renal cell damage and the subsequent development of AKI in malaria patients. Although, the results show clear statistical significance, a larger sample size and serial determination of urinary NF-κB p65 levels can further address its benefit over serum Cr level.

NF-κB p65 levels in urinary sediment cells showed a significant positive correlation with serum Cr on the day of admission and day 7 post-treatment. It has been reported that increased serum Cr was the major factor and an important predictor for mortality among malaria patients with AKI, and that early diagnosis and prompt management including dialysis can reduce mortality [26]. A positive correlation was found between NF-κB p65 levels and parasite density on day of admission. This finding can be associated with a higher risk of developing malaria AKI, as previous study has shown that patients with parasitaemia >5%, corresponding to 250,000 PRBCs per μl were at higher risk of developing malaria AKI than those with lower parasitaemia [27]. In addition, NF-κB p65 showed a significant positive correlation with microscopic WBC on day of admission and day 7 post-treatment. The findings correlate with the activation of NF-κB p65 reported in the PBMCs of malaria patients [24].

## Conclusions

The levels of NF-κB p65 in urinary sediment cells reflect the degree of damage in renal tubular epithelial cells in malaria patients. Therefore, the urinary level of NF-κB p65 has a potential role as a disease biomarker in estimating damage to renal tubular epithelial cells and subsequent progression of AKI among complicated *P. falciparum* malaria patients. It will be useful to investigate the NF-κB p65 expression level in specific cell types in urinary sediments, to further explore the exact precursor cells of NF-κB and establish its role in the pathogenesis of AKI in malaria.

## Competing interests

The authors declare that they have no competing interests.

## Authors’ contributions

CP performed the laboratory work, obtained clinical data and drafted the manuscript. PV designed the experiments, recruited the patients, analysed the data, and revised the final manuscript. Both authors have approved the final version of this manuscript.
